# Useful Recipes for the Household

**Published:** 1871-04

**Authors:** 


### Domestic Medicine.

### USEFUL RECIPES FOR THE HOUSEHOLD.

RHEUMATISM.—A very simple remedy for rheumatism of the extremities, and one that very often gives great relief is, to take a large piece of thick flannel, sprinkle it well with finely pulverised sulphur, and then bind snugly about the limb with the sulphur next the skin.

CORNS.—Every body has a remedy for corns; here's another:—Macerate the tender leaves of ivy in strong vinegar for eight or ten days, then apply to the corns by means of cloths or lint saturated with the liquor. In a few days the corns will drop off.

TO STOP NOSE-BLEEDING.—Press the lip tightly to the jaw with the thumb, held firmly just under and to the right (or left) of the nostril. A branch of the facial artery may be felt in this locality, on either side, and when the circulation is arrested the bleeding in the nose stops.

TOOTH-ACHE.—If the tooth contains a cavity which can be easily reached, fill it with *sugar of lead.* Allow it to remain a few minutes, then wash it out with warm water, being careful to remove all of it. This is the most prompt relief for tooth-ache—save the forceps—with which we are familiar.

MUMPS.—No external application is necessary. Keep the patient within doors—avoid drafts of cold air—give a mild cathartic, and then administer *muriate of ammonia* every three hours, in twenty to forty grain doses. It will cure almost any case of mumps in two or three day's time.

INFLAMMATION AND ITCHING OF THE SKIN.—In acute inflammation of the skin, attended with severe itching and a moist irruption, the following powder rubbed over the parts will at once allay the itching:

Take:

Powdered starch, six drachms,
Oxide of zinc, three drachms,
Powdered camphor, half drachm,
Cochineal, one grain.

Mix thoroughly, and keep in a glass stoppered bottle.

FACIAL NEURALGIA.—Procure a half ounce of the oil of peppermint, and, with a camel's-hair brush, paint the parts of the face where the pain is felt. We have found it an excellent application in all forms of pain in the face. A drop applied to the cavity of an aching tooth, and confined there with a pellet of cotton, will arrest the pain.

DEADLY CHEESE.—Those fascinating little foreign cheeses which are so neatly done up in tin foil, and which tickle the palates of our epicures, are not the healthiest morsels in the world. The tin foil contains lead; and "the lactic acid of the cheese, favored by the condition of moisture, leads to contamination of the outer layers with that poisonous metal." So chemists tell us, and they are supposed to know—therefore avoid this tempting little morsel, and take your lead in some less dangerous form.

POISON BY IVY.—Some persons are very susceptible to the poison of this vine. It causes the skin to break out in little vesicles which smart, itch, and burn intolerably. If the parts affected are rubbed or scratched, sores are produced which are loath to heal. Often the face is so swollen as to entirely close the eyes. A remedy for all this mischief will be found in the following:—

Take a quarter of a pound of quick-lime, dissolve it in water, let it stand until cold, then smear the parts with it. Allow it to remain twenty minutes, wash off in warm water, then smear with sweet oil. Three or four applications of this kind will cure the most aggravated case.

GREEN VEILS.—We frequently see little children in their carriages upon the street, with green veils tied over their heads and faces. A child will always take the folds of the veil in its mouth, when it can, and will often extract the green coloring matter with its lips. Children and even grown people, have sore mouths and faces from this cause, which are frequently difficult to heal. The coloring matter in the green veil contains arsenic, which, when placed in contact with a delicate surface like the lips, or a pimple upon the face, will cause an ulcer that is troublesome to heal. Ladies sometimes have sore hands from wearing green gloves, when they innocently attribute the difficulty to another cause, thinking that their hands are chapped.—Green colored wearing apparel of any character, should never be worn next the skin.

ERYSIPELAS.—This name has a fearful sound to most persons. The disease is not as serious a one as people are in the habit of believing. It generally attacks the face, scalp or neck. Simple erysipelas is an inflammation of the skin and the tissue immediately under it. It is attended with considerable swelling and a burning, tingling heat. Sometimes little vesicles appear, which contain an amber-colored liquid, and which soon rupture, giving place to dark-colored crusts or scabs. We give this short description of the disease for the reason that most people are in the habit of calling every little sore or fester about the face, erysipelas. By the above description you will have no difficulty in recognising the disease. And now for the remedy:—Paint the swollen parts thoroughly, with *tincture chloride of iron.* Do this by means of a feather or camel's-hair pencil. After you have coated the parts until it presents a deep orange color, commence taking twenty drops of the same medicine, in water, every four hours. Continue the paintings and the internal administration of the medicine, at intervals of four hours, until the swelling, inflammation and pain subside, which in ordinary cases, should occur in from twenty-four to forty-eight hours.

HAIR DYES.—A French journal asserts that seven per cent. of lunatics are made so by the employment of hair dyes. We disbelieve the assertion for the reason that we think most persons are lunatics just before beginning to dye their hair. We have frequently shown the danger of using the very many "hair restorers" with which the market is flooded; but, as many people *will* dye their hair, even if it kills them, we offer them a harmless way of doing it. The dye must be prepared in the fall of the year, and kept for use. Procure a half bushel of black walnut hulls. They must be taken when nearly, or quite ripe, and the hull is yet green. Place them in a stone jar and cover them with soft water. Allow them to macerate for one week, then strain the liquor from them, when it is ready to apply to the hair. This must be done with a brush, and with care, so as not to touch the skin, as it will color that also. After the hair or beard is so colored, the following dressing can be applied, which will improve its appearance:

Take equal parts of glycerine and water, and to one pint of the mixture add a teaspoonful of lac sulphur and a few drops of oil of bergamot. Shake this thoroughly every time before using, and apply to the hair as a dressing, once a day. This is a harmless and effectual way of coloring the hair for those who *will* persist in so disguising themselves.

---

COUGHS AND COLDS.—This is the season of the year when almost every one suffers more or less from coughs or colds. People *will* be careless about dress. As the spring weather approaches, they are apt to throw off their thick clothing too soon, and many are foolish enough to remove their underclothing after experiencing a few days of warm and summer-like weather. Soon the air changes to cold, and even to a frigid temperature, when such persons suffer from catarrh and "colds in the head," or a distressing cough. We give below several simple, safe, and effective formulæ which may be used with benefit.

Take:
Syrup of squills,
Syrup of ipecacuanha,
Camphorated tinct. of opium, of each, one ounce.

Mix them together; an adult may take a teaspoonful every two or three hours. This medicine will promote expectoration when the cough seems dry and "tight."

The following formula may be used with great comfort to one suffering with cold in the head, sneezing, and a tickling sensation about the throat and faucies:

Take:
Bal. Copaiba,
Bal. of tolu,
Powdered gum arabic, of each ½ ouncce,
Water, six ounces,
Aromatic sulphuric acid, 20 drops.

Mix, and shake the bottle thoroughly. Take a teaspoonful every three hours.

In the more advanced stage of bronchitis, with a constant disposition to cough night and morning, with little or no expectoration, use the following:

Take:
Syrup of wild cherry bark, 3 fluid oz.,
Syrup of tolu, 1 fluid oz.,
Prussic acid, diluted, 15 drops.

Mix, shake thoroughly, and for an adult give a dessertspoonful every three hours.

Your druggist can put up any of the above formulæ, which will be found safe and effective.

